# Efficacy and safety of subthreshold micropulse laser compared with threshold conventional laser in central serous chorioretinopathy

**DOI:** 10.1038/s41433-019-0692-8

**Published:** 2019-11-29

**Authors:** Zuhua Sun, Ying Huang, Chaochao Nie, Zhijie Wang, Junqing Pei, Bing Lin, Rong Zhou, Junyan Zhang, Victor Chong, Xiaoling Liu

**Affiliations:** 1 School of Ophthalmology & Optometry and Eye Hospital, Wenzhou Medical University, 270 Xueyuan Road, Wenzhou, Zhejiang China; 2 Bothwin Clinical Study Consultant, Redmond, WA USA; 3 Optegra Eye Hospital, London, UK; 4 Royal Free Hospital, London, UK

**Keywords:** Eye diseases, Physiology

## Abstract

**Purpose:**

To compare the efficacy and safety of subthreshold micropulse laser (SML) with threshold conventional laser (TCL) in central serous chorioretinopathy (CSC).

**Methods:**

Prospective, randomized, double-masked, non-inferiority, 12-week clinical trial. Patients were randomly assigned 1:1 to SML group or TCL group. Patients in the SML group were treated with 577 nm micropulse laser. The spot size was 160 µm, the duty cycle was 5% and exposure time was 0.2 s. The power was 50% threshold tested. Patients in the TCL group were treated with 577 nm continuous laser. The power was 100% threshold tested. The primary outcome was the mean change in best-corrected visual acuity (BCVA) at week 12, with a non-inferiority limit of five letters on the Early Treatment Diabetic Retinopathy Study (ETDRS) visual acuity charts.

**Results:**

Eighty-eight patients were enroled. Seventy-seven patients were male. Forty-four patients were in SML group and 44 in TCL group. At week 12, SML was equivalent to TCL with a gain of 6.23 ± 8.59 and 6.61 ± 6.35 letters, respectively, (SML–TCL difference: −0.38 letters; 95% confidence interval (CI):−3.58–2.81; *P*_non-inferiority_ = 0.0026). There was no statistically significant difference between the two groups (*t* = 0.240, *P* = 0.811). At week 12, the proportion of patients whose SRF had been totally absorbed was 63.63 and 81.82% respectively for SML and TCL groups. There was no statistically significant difference between the two groups (χ^2^ = 3.67, *P* = 0.056).

**Conclusions:**

Both SML and TCL can improve visual acuity in CSC. SML was non-inferior to TCL in the improvement of BCVA.

## Introduction

Central serous chorioretinopathy (CSC) is a relatively common early-onset disease characterized by serous detachment of the neural retina with dysfunction of the choroid and retinal pigment epithelium (RPE). One or more focal leakages of RPE decompensation that have been identified by mid-phase fundus fluorescein angiography (FFA) are considered the principal sources of subretinal fluid (SRF) [1, 2].

Conventional laser photocoagulation and photodynamic therapy (PDT) are effective methods for treatment of CSC [3, 4]. Conventional laser photocoagulation is a precise application of laser treatment at a suprathreshold power that creates grade I burns. It is an effective and routine intervention in CSC. Threshold conventional laser (TCL), which may be safer [5], uses less power than the grade I burns. However, both conventional laser and TCL are not appropriate for juxtal or subfoveal leakage because they may cause central or paracentral scotomas, contrast sensitivity loss, accidental foveal damage, retinal distortion and choroidal neovascularization (CNV) as well as atrophic scars on the RPE [6–8]. PDT uses a laser to activate a dye locally, leading to choroidal vascular thrombosis [9]. PDT is an option not only for chronic CSC with diffuse leakage but also for acute CSC with leakage within or close to the fovea [3, 10–12]. While it has not been approved for health insurance in China, and it is very expensive. Under these circumstances, PDT has been limited in China.

Unlike conventional laser, a subthreshold micropulse laser (SML) is non-damaging, the para-fovea or fovea can be directly treated, and treatment can be repeated without detectable structural or functional damage [13]. SML involves a series of repetitive laser pulses with an interval. SML have been used in the treatment of a wide range of retinal disease in clinical studies and are commercially available and FDA approved [13], meaning that SML can be considered as an alternative treatment for CSC.

Although SML has been reported in the literature treatment of CSC, the parameters used have been controversial. Several studies using 810 nm micropulse lasers have demonstrated efficacy in the treatment of CSC [14–20]; however, only a few studies have been conducted using 577 nm micropulse lasers, and all were done retrospectively [21–24]. The 577 nm wavelength occurs outside the absorption spectrum of retinal xanthophyll and has major advantages, such as peak absorption of oxyhaemoglobin, minimal xanthophyll absorption in the macula and better penetration, potentially allowing for treatment close to the fovea [25, 26]. This study is the first prospective, randomized controlled trial comparing the efficacy and safety of 577 nm SML with 577 nm TCL in the treatment of CSC.

## Subjects and methods

This was a single-centre, prospective, randomized, double-masked, non-inferiority, 12-week clinical trial comparing the efficacy and safety of SML with TCL in the treatment of CSC. The study was registered with ClinicalTrials.gov (identifier no. NCT02735213). All patients were recruited from the School of Ophthalmology & Optometry and Eye Hospital, Wenzhou Medical University. The study protocol was approved by the ethics committee of School of Ophthalmology & Optometry and Eye Hospital, Wenzhou Medical University. The study was carried out in compliance with the Declaration of Helsinki and the International Conference on Harmonization Guidelines for Good Clinical Practice. All patients provided written informed consent to participate in this trial. The data described here were collected between April 2016 and February 2017.

### Participants

**Fig. 1:**
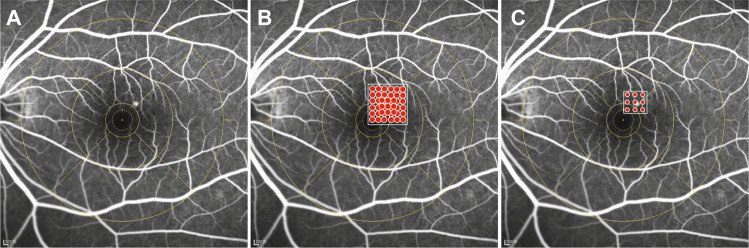
**a** FFA of mid-phase showed the leakage located at the ring 2. **b** Stimulated image of the subthreshold micropulse laser mode. The white square frames represented the treatment area. The red dots represented the laser points. **c** Stimulated image of the threshold conventional laser mode.

Patients ≥ 18 years with CSC causing SRF involving the centre of the macula within 6 months were eligible for enrolment if central retinal thickness (CRT) measured by spectral-domain optical coherence tomography (SD-OCT) (Spectralis; Heidelberg Engineering, Heidelberg, Germany) was ≥250 μm, best-corrected visual acuity (BCVA) was at least 64 Early Treatment Diabetic Retinopathy Study (ETDRS) letters (20/50 Snellen equivalent), and the leakage points on FFA were limited in ETDRS ring 2 or 3 (Fig. 1a). Only one eye from each patient was included.

Exclusion criteria were: PDT and/or laser treatments in the study eye within 3 months prior to enrolment, and the presence of other retinal lesions such as CNV, polypoidal choroidal vasculopathy or age-related macular degeneration.

Ninety-two patients provided written informed consent. Eighty-eight patients were enroled and randomized 1:1 into the SML or TCL group according to a predetermined randomization scheme provided by a designated, masked statistician. A secure, computer-generated randomization schedule was maintained in concealed envelopes by a study-group member who did not participate in enrolment. Concealed envelopes were revealed by treatment physicians only after eligibility for enrolment had been confirmed and before treatment were given to the patients.

### Treatments

Patients in the SML group were treated with the Supra Scan 577 nm micropulse laser mode (Quantel Medical SubLiminal laser). The laser parameter was set to 5% duty cycle, spot size was 160 μm, 9-spot matrix without spacing between two spots was used (Fig. 1b). On the micropulse mode, the threshold power was titrated at the arch area. SML was performed on the leakage areas on a mid-phase FA image with the 50% threshold power [9, 21]. The total laser burns made were 150–200.

Patients in the TCL group were treated with the Supra Scan 577 nm continuous laser mode (Quantel Medical, France). The laser parameter was set to the time was 0.05 s, spot size was 100 μm, and 9-spot matrix with one burn space (Fig. 1c). On the conventional laser mode, the threshold power was titrated near the arch area. TCL was then performed on the leakage areas on a mid-phase FA image with the threshold power tested. The laser burns made were 18–27.

**Fig. 2:**
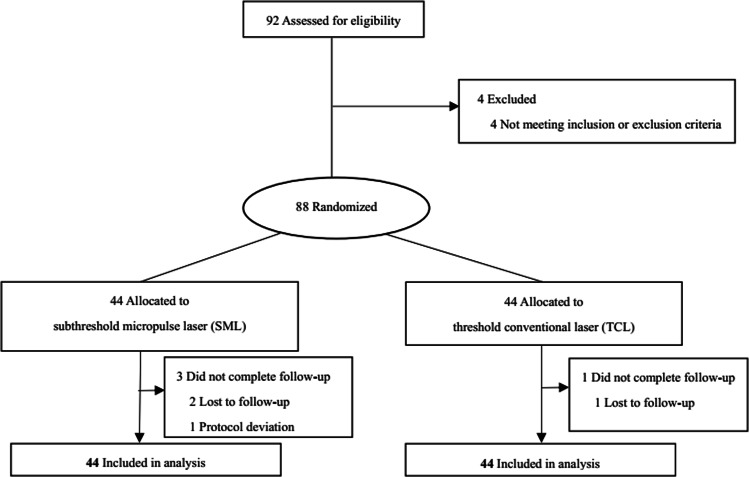
Study follow-up flow chart. SML Subthreshold micropulse laser, TCL threshold conventional laser.

All the treatments were performed by a single investigator. Prior to treatment, all patients underwent ophthalmic examinations, including BCVA, anterior segment examination, dilated fundus examination, fundus photography (FP) (Canon, CR-1 Mark II, Japan), SD-OCT, FA and indocyanine green angiography (ICGA) (Spectralis, Heidelberg Engineering, Heidelberg, Germany). Patients were re-assessed at 3, 7 and 12 weeks after treatment. BCVA, fundus and OCT examinations were performed. The follow-up flowchart was outlined in Fig. 2.

### Outcome measurements

The primary efficacy outcome was change in BCVA from baseline to week 12. The secondary were the proportion of patients who had totally absorbed SRF at week 12, changes in BCVA, CRT and central choroidal thickness from baseline to week 3, 7 and 12, respectively. Additional outcomes included the proportion of eyes that gained ≥5 ETDRS letters in BCVA from baseline to week 12, as well as the proportion of eyes that accepted retreatment at week 12. Safety assessments included laser-induced scarring detection on FP at week 12, and any other ocular or systemic event during the 12 weeks of follow-up.

BCVA was assessed following the ETDRS protocol [27] by the certified masked optometrist. SRF, CRT and central choroidal thickness were assessed using SD-OCT by two qualified masked technicians. The OCT examination included 25 sections, each of which comprised nine averaged scans and which were obtained in an area of a 6 mm × 6 mm square centred on the fovea. Central choroid thickness was measured using EDI mode on SD-OCT. The leakage located at ring 2 or 3 on the FA image was assessed following the ETDRS protocol [28]. All of them except the physician who administered the laser treatment were unaware of the patient’s treatment assignment.

RPE atrophy was specified as RPE defect, pigmentation was specified as more pigment than usual and migration was specified as pigment displacement by FP.

### Statistical analysis

This study was designed as a non-inferiority trial comparing the two groups. For the primary outcome, non-inferiority limit for the difference between two groups in the mean change in BCVA at week 12 was five letters [29]. Assuming a standard deviation (SD) for changes in BCVA of ten letters, an overall one-sided type I error rate of 0.025, a rate of loss to follow-up of 20% and a power of 80%, we determined that a sample of 35 patients per group, adjusted to 44 each.

A one-tailed statistical test for non-inferiority between the two groups was performed. The primary analysis followed the intention-to-treat principle. Missing data were imputed using the last-observation-carried-forward method. Statistical analysis of the data between baseline and follow-up in each group was performed. Means ± SD are reported. *P* values and confidence intervals (CI) are two sided. *P* values less than 0.05 were considered statistically significant. Mean values between groups in continuous variables were compared using independent *t*-tests. Changes in BCVA, CRT and central choroidal thickness of both groups from baseline to follow-up were compared using paired *t*-tests and the Wilcoxon rank-sum (Mann–Whitney) test. Chi-square was used to compare categorical data between the two groups.

## Results

Eighty-eight patients were randomized into the SML group (*n* = 44) and TCL group (*n* = 44). Minor differences in baseline clinical characteristics existed between the two groups, as outlined in Table 1. During the study, no patient was using any oral, topical, inhaled or injected corticosteroids. Four patients dropped out of the study without explanation: three in the SML group and one in TCL group. The proportion of patients who completed the study was 95%.

**Table 1:** Patient demographics and baseline characteristics.

	SML (*n* = 44)	TCL (*n* = 44)	*t* or χ^2^ or *z*	P1	P2
Sex					
Male, *n* (%)	35 (79.55)	42 (95.45)			
Female, *n* (%)	9 (20.45)	2 (4.55)	5.091	0.024*	
Age (years)					
Mean ± SD	44.41 ± 8.71	44.68 ± 6.77	0.164	0.87	
Median (IQR)	44 (39–51.5)	45 (40–48)	0.322		0.748
Duration (weeks)					
Mean ± SD	10.50 ± 8.22	10.20 ± 8.22	−0.169	0.867	
Median (IQR)	7.5 (4–18)	6.5 (4–18)	−0.324		0.746
BCVA (ETDRS letters)					
Mean ± SD	76.60 ± 7.00	77.84 ± 7.70	0.159	0.874	
Median (IQR)	77 (73–80)	77.5 (72–82)	0.209		0.835
CRT (μm)					
Mean ± SD	398.50 ± 92.92	407.49 ± 133.55	0.367	0.715	
Median (IQR)	381 (332–445.75)	374 (324.5–458)	−0.142		0.887
Leakage location					
Ring 2, *n* (%)	35 (79.55)	34 (77.27)			
Ring 3, *n* (%)	9 (20.45)	10 (22.73)	0.067	0.796	
Leakage point					
Single, *n* (%)	35 (79.55)	35 (79.55)			
Multiple, *n* (%)	9 (20.45)	9 (20.45)	0	1	
Primary/recurrent					
Primary, *n* (%)	41 (93.18)	37 (84.09)	1.805	0.179	
Recurrent, *n* (%)	3 (6.82)	7 (15.91)			
Prior laser treatment *n* (%)	1 (2.27)	4 (9.09)	0.848	0.357	
Systemic steroid *n* (%)	0 (0)	2 (4.55)	0.512	0.474	

Full analysis set. P1 represented the *P* value of the independent *t*-test result or χ^2^. P2 represented the *P* value of the Wilcoxon rank-sum (Mann–Whitney) test

*SML* subthreshold micropulse laser, *TCL* threshold conventional laser, *SD* standard deviation, *IQR* interquartile range, *BCVA* best-corrected visual acuity, *ETDRS* Early Treatment Diabetic Retinopathy Study, *CRT* central retinal thickness

*Significant *P* value (<0.05)

**Fig. 3:**
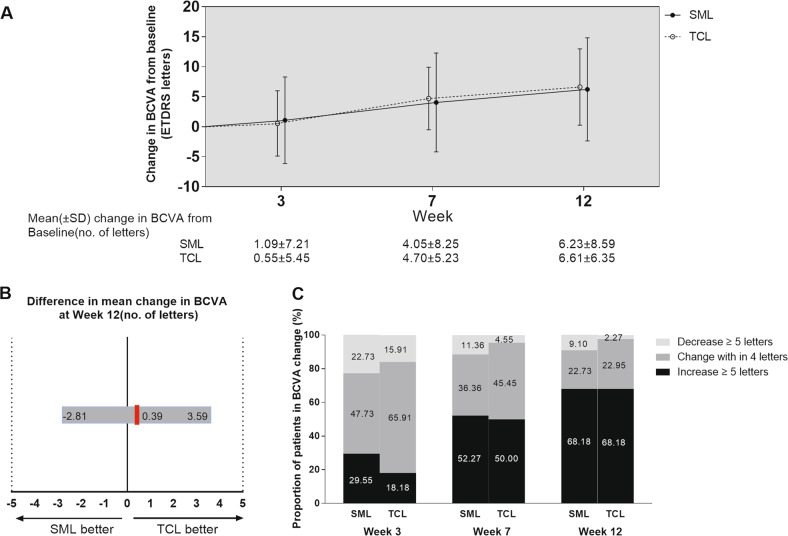
**a** Mean change in BCVA. **b** Differences in BCVA change from baseline to week 12 between the two groups. The red vertical lines indicated the mean difference between the two groups, and the grey bar was the 95.0% CI. CI within −5 and +5 letters (dashed vertical lines) indicated that the two groups were equivalent. A lower limit of the 95.0% CI with a value above −5 showed that SML was non-inferior compared with TCL. **c** Proportions of patients with BCVA change from baseline to week 12. BCVA best-corrected visual acuity, CI confidence interval, SML subthreshold micropulse laser, TCL threshold conventional laser.

Visual acuity improved from baseline to 12 weeks in both groups (Fig. 3a). At week 12, the changes in BCVA were not statistically significant between the groups (*P* = 0.811, *t*-test). SML was equivalent to TCL with a gain of 6.23 ± 8.59 (*P* < 0.001) and 6.61 ± 6.35 (*P* < 0.001) letters, respectively, in the full analysis set (SML–TCL difference, −0.38 letters; 95% CI, −3.58–2.81; *P*_non-inferiority_ = 0.0026) (Fig. 3b) and with a gain of 5.55 ± 8.45 and 5.45 ± 5.26 respectively by intention-to-treat analysis (SML–TCL difference, 0.09 letters; 95% CI, −2.89–3.07; *P*_non-inferiority_ = 0.0005). At week 12, the mean BCVA was 82.82 ± 7.71 and 83.45 ± 6.40 letters in the SML group and in the TCL group, respectively. The proportion of patients who gained ≥5 letters from baseline to week 12 was 68% in the SML group, the same as that in the TCL group (*P* = 1.0, Chi-square test) (Fig. 3c).

**Fig. 4:**
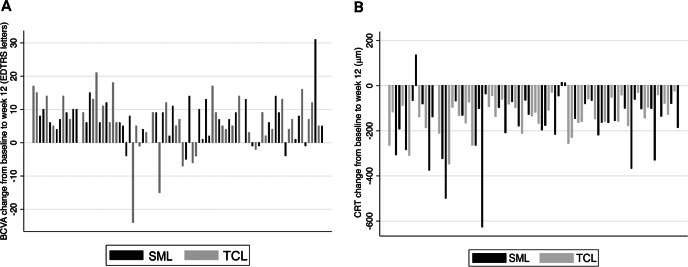
**a** Waterfall plots of BCVA changes from baseline to week 12 for individual patients. These plots showed that all the BCVA scores improved except six patients in SML group and seven patients in TCL group. **b** Waterfall plots of CRT changes from baseline to week 12 for individual patients. These plots showed that all the CRT thicknesses decreased from baseline except three patients in the SML group, but none in TCL group. BCVA best-corrected visual acuity, CRT central retinal thickness, SML subthreshold micropulse laser, TCL threshold conventional laser.

At week 12, all the BCVA scores were over baseline except six patients in the SML group and seven in the TCL group (Fig. 4a). BCVA lost 24 letters from baseline in one patient was because of secondary CNV after the TCL treatment. BCVA lost 15 letters from baseline in one patient in the TCL group because the CRT was only 203 μm, which was thinner than normal. BCVA lost within 10 letters in the other 11 patients was either because of persistent SRF or because of disorder of the photoreceptor layer.

The proportion of patients whose SRF had been totally absorbed at week 12 was 63.63% for the SML group, and 81.82% for the TCL group. There was no statistically significant difference between the two groups at week 12 (*P* = 0.056).

The mean reduction of CRT from baseline to week 3 was 101.39 ± 97.03 μm in the SML group and 140.92 ± 113.68 μm in the TCL group. Mean reduction at week 7 was 126.47 ± 90.05 μm in the SML group and 152.66 ± 131.50 μm in the TCL group. The mean reduction at week 12 was 131.66 ± 79.26 μm in the SML group (*P* < 0.001, *t*-test) and 163.00 ± 136.90 μm in the TCL group (*P* < 0.001). There was no statistically significant difference between groups (*P* = 0.192). At week 12, CRT decreased from baseline except for three patients in the SML group; all members of the TCL group showed a decrease (Fig. 4b).

Central choroidal thickness at baseline was not available for two patients, meaning that data from 86 patients were analysed. The mean reduction of central choroidal thickness from baseline to week 12 was 22.98 ± 54.94 μm in the SML group and 42.94 ± 46.08 μm in the TCL group (*P* = 0.009; *P* < 0.001; *t*-test, respectively). There was no statistically significant difference between groups in the change of central choroidal thickness at week 12 (*P* = 0.071).

### Safety

Secondary CNV occurred in one patient in the TCL group. No RPE atrophy, pigmentation, migration or systemic adverse events were observed in the other 87 patients. No laser-induced scarring was detected and only mild RPE depigmentation was detected in 12% patients in the SML group at week 12 by FP images, whereas laser-induced changes were detected in 33% of patients in the TCL group. The difference between the groups was statistically significant (*P* = 0.032).

## Discussion

Comparing 577 nm SML and 577 nm TCL, the effects of SML on visual acuity at 12 weeks were non-inferior to TCL treatment. The proportion of patients who gained at least five letters, the proportion of total SRF absorption and the retreatment rate were practically the same for each group.

Low power (5% duty cycle, 50% power of threshold) and high density (150–200 confluent spots in the leakage area) SML for CSC were used in this study. Not only can this effectively accelerate recovery, but it can also improve BCVA. The mechanism of action of SML for CSC was hypothesised as biological activation of the decompensated RPE cells in the leakage area by subtle thermal stress without causing RPE or photoreceptor cell death.

In this study, SML was non-inferior to TCL in terms of BCVA improvement at week 12. Interestingly, the curve of the change in BCVA from baseline differed from the curve of the change in CRT from baseline, which dropped sharply at 3 weeks after SML. The improvement of BCVA was slower than the reduction of CRT. It is likely that the photoreceptor cells need time to recover after the neural retina reattachment and total absorption of SRF have taken place.

SML activated the RPE without damaging RPE or photoreceptors. There was a number of other interventional case series showing complete resolution of SRF were between 55 and 79% of patients treated with SML and at least partial resolution in 75–100% of patients over 2–14 months’ follow-up [8, 18, 20, 23, 30, 31]. Scholz et al. reported that only 24% of patients dried up totally in the retrospective study with 577 nm SML after the treatment of chronic CSC [21]. SRF had been totally resolved in about 50% patients at week 3 and 65% at week 12 after the 577 nm SML treatment in our study, which was better than Scholz. This more favourable outcome is perhaps due to short disease duration (within 6 months); most patients were also naive in this study. In the TCL group, the proportion of SRF had totally resolved was much higher than in the SML group, although this difference was not statistically significant. If complete SRF absorption after a single treatment is the major aim, TCL 577 nm is the preferred treatment.

The effect of SML treatment for CSC in the different investigations varies according to different heterogeneous inclusion criteria, laser parameters and laser spot performance area. Some authors adjust laser power upward to the minimum threshold value for a visible burn in a continuous wave mode and then switch the apparatus to micropulse mode [15, 18, 23]. Other researchers have also used the micropulse mode for power titration and applied 50–80% of the minimum threshold power to cause a barely visible burn [19, 22]. The latter power adjustment method was used in this study. Some authors determine the area to be treated as active leakage on mid-phase FA or hyperfluorescent areas on mid-phase ICGA images [14, 21, 23, 32]. Malik et al. determine the area to be treated as limited to one disk diameter surrounding fluorescein leakage or the areas of serous elevation as determined by clinical examination and OCT [31]. Kim et al. delivered laser shots over the entire area of CSC, including the leaking point on FA and the foveal centre, and also on the normal retina around the borderline area of serous retinal detachment [22]. The area to be treated was determined as active leakage on mid-phase FA or hyperfluorescent areas on mid-phase ICGA images in this study. Further studies are needed to determine a standard power titration protocol and treatment area in SML applications.

SRF had totally resolved in about two thirds of patients, with no laser-induced scarring, pigmentation, CNV or atrophy, at 12 weeks after SML treatment. This indicates that the parameters including laser spots and density of SML for CSC were effective and safe in this study. For the remaining third of patients, we speculate that the intensity of the laser was not enough to fully activate the decompensated RPE. The retreatment rate was 11% at week 12 in the SML group, which was similar to the TCL group. The result was better than in Scholz’s study, which had a retreatment rate of 55% (39% twice and 16% three times) [21]. This could be due to patients who were chronic, including some persistent SRF after PDT in Scholz.

No systemic adverse events were observed in each group. Secondary CNV in one patient and some laser-induced scarring was detected in the TCL group. No scarring was detected in the SML group, suggesting that SML was safer than TCL.

The main limitation of this study is that the follow-up period is relatively short with only 12 weeks. Recurrences are relatively common in CSC, so a longer follow-up period might add much value in the future studies. Another limitation is the absence of an untreated control group or a half-dose PDT group, so it was difficult to determine whether the results were due to the laser treatment or spontaneous resolution because the disease durations in this study were all within 6 months.

In conclusion, the 577 nm SML was effective and safe in CSC patients, and it was non-inferior to the 577 nm TCL in the improvement of BCVA. In the cases where leakage is very near to the fovea, SML may be preferred to TCL.

### Summary

#### What was known before

- TCL is usually performed in the treatment of CSC

#### What this study adds

- SML is non-inferior to TCL in CSC treatment
